# Implementing the United Kingdom’s ten-year teenage pregnancy strategy for England (1999-2010): How was this done and what did it achieve?

**DOI:** 10.1186/s12978-016-0255-4

**Published:** 2016-11-22

**Authors:** Alison Hadley, Roger Ingham, Venkatraman Chandra-Mouli

**Affiliations:** 1 Teenage Pregnancy Knowledge Exchange, Faculty of Health and Social Sciences, University of Bedfordshire, Luton, Bedfordshire LU1 3JU UK; 2 University of Southampton, Southampton, UK; 3 World Health Organisation, Geneva, Switzerland

**Keywords:** Teenage pregnancy, Health strategy, England, Sexual health, Adolescents

## Abstract

**Background:**

In 1999, the UK Labour Government launched a 10-year Teenage Pregnancy Strategy for England to address the country’s historically high rates and reduce social exclusion. The goal was to halve the under-18 conception rate. This study explores how the strategy was designed and implemented, and the features that contributed to its success.

**Methods:**

This study was informed by examination of the detailed documentation of the strategy, published throughout its 10-year implementation.

**Results:**

The strategy involved a comprehensive programme of action across four themes: joined up action at national and local level; better prevention through improved sex and relationships education and access to effective contraception; a communications campaign to reach young people and parents; and coordinated support for young parents (The support programme for young parents was an important contribution to the strategy. In the short term by helping young parents prevent further unplanned pregnancies and, in the long term, by breaking intergenerational cycles of disadvantage and lowering the risk of teenage pregnancy.). It was implemented through national, regional and local structures with dedicated funding for the 10-year duration. The under-18 conception rate reduced steadily over the strategy’s lifespan. The 2014 under-18 conception rate was 51% lower than the 1998 baseline and there have been significant reductions in areas of high deprivation. One leading social commentator described the strategy as ‘The success story of our time’ (Toynbee, The drop in teenage pregnancies is the success story of our time, 2013).

**Conclusions:**

As rates of teenage pregnancy are influenced by a web of inter-connected factors, the strategy was necessarily multi-faceted in its approach. As such, it is not possible to identify causative pathways or estimate the relative contributions of each constituent part. However, we conclude that six key features contributed to the success: creating an opportunity for action; developing an evidence based strategy; effective implementation; regularly reviewing progress; embedding the strategy in wider government programmes; and providing leadership throughout the programme. The learning remains relevant for the UK as England’s teenage birth rate remains higher than in other Western European countries. It also provides important lessons for governments and policy makers in other countries seeking to reduce teenage pregnancy rates.

## Plain English summary

The UK Labour Government’s 10-year teenage pregnancy strategy for England is one of the few examples of an intervention which has successfully reduced teenage pregnancy rates. The strategy, launched in 1999, was the first attempt by the government to implement a comprehensive, evidence based programme with sufficient time, funding and leadership to have an impact. Nationally led and locally delivered, the strategy had four themes: joined up action by national and local government; better prevention through improved sex and relationships education and young people’s access to effective contraception; a national campaign to reach young people and parents; and coordinated support for young parents. Since the start of the strategy the under-18 conception rate in England has fallen by 51%, with significant reductions in deprived areas. This paper describes the detail of how the complex strategy was implemented over the 10 year period and discusses six key features which are identified as being fundamental to its success: creating an opportunity for concerted action; developing an evidence based strategy; establishing structures and guidance for effective implementation; regularly reviewing progress; embedding strategy actions in wider government programmes; and providing government leadership throughout the 10 year programme.

The learning from the strategy remains relevant for UK policy and practice as, despite the substantial decline, England’s teenage birth rate remains higher than in other Western European countries. The paper also provides important lessons for governments and policy makers in other countries seeking to reduce teenage pregnancy rates.

## Background

The Teenage Pregnancy Strategy (TPS) was the first comprehensive approach by any UK government to reduce England’s high teenage pregnancy rates. The rate had been, for many years, higher than other western European countries and outcomes for young parents and their children were disproportionately poor. The strategy was launched in 1999 with a goal of halving the under-18 conception rate. By 2014, the rate had dropped 51% [1]. The purpose of this study was to explore how the strategy was designed and implemented to achieve its success. This paper complements two previously published articles. The first [2] presents an overview of the strategy within the WHO ExpandNet framework; the second [3] confirms the impact of the strategy by combining detailed analysis of the outcomes along with the data from the Natsal-2 and Natsal-3 studies. Although there is inevitably some overlap of material, this new paper provides significantly more detail, serving to illustrate the complexities involved in implementing a strategy that achieved such a high level of change, refers how to access key documentation, and identifies six key features which were fundamental to its success.

## Methods

This study was informed by reviewing the detailed documentation of the strategy. This included the original rationale and action plan of the strategy, the annual reports to parliament by the Independent Advisory Group on the progress of the strategy and Government’s responses, and a range of guidance published to support implementation. The written documentation was supplemented by the personal experiences of two of the authors; one (AH) who led the implementation and the other who was the research lead (RI) on the advisory group.

## Results

### The early years

The 10-year strategy was developed over an 18 month period by the then Labour Government’s newly established Social Exclusion Unit (SEU), informed by a detailed review of the international and national evidence, interviews with national stakeholders, and consultations with professional organisations, NGOs and young people. It was published in 1999 with a 30-point action plan, across four themes: joined up action at national and local levels; better prevention for both girls and boys through improved sex and relationships education and access to effective contraception; a national communications campaign to reach young people and their parents and carers; and coordinated support for young parents [4]. The support programme was an important contribution to the prevention strategy, but the details are not reported in this article (but see [5]).

The headline target was to halve the under-18 conception rate between 1998 (the selected baseline year) and 2010.

A Teenage Pregnancy Unit (Unit) was established with cross-government funding to lead the implementation of the strategy, with a team combining civil servants and external experts drawn from the statutory sector and NGOs. The Department of Health held the national ministerial lead; however, to signal government’s collective responsibility for the strategy’s implementation, a minister for teenage pregnancy was appointed in each department and an inter-departmental group of officials met quarterly with TPU to monitor progress. Regional and local structures mirrored the national structure, with Regional Teenage Pregnancy Coordinators (regional coordinator), local Teenage Pregnancy Coordinators (local coordinator) and local Teenage Pregnancy Partnership Boards (partnership boards).

An Independent Advisory Group on Teenage Pregnancy (Advisory Group) consisting of expert stakeholders was appointed to monitor implementation of the strategy, to advise ministers on a regular basis and to submit an annual report to government. The strategy was supported by a group of NGOs and a national inter-faith forum [6].

An annual local implementation grant was provided to each of the 150 local government areas (average population size – 375,000, range 34,000 to 1,100,000), averaging 300–400,000 GBP. The grant was intended to supplement, not replace, mainstream funding with conditions on spending mandating the appointment of the local coordinator and partnership board, developing local plans and providing an annual report on progress [6].

To support local government and partners to work together, the strategy action plan tasked the Unit to publish a range of guidance on the following topics:

- ➢ sex and relationships education in schools [7];
- ➢ young people friendly sexual and reproductive health services [8];
- ➢ improving the uptake of sexual and reproductive health (SRH) advice by boys and young men [9];
- ➢ improving the uptake of SRH advice by black and minority ethnic young people [10];
- ➢ teenage pregnancy and diverse communities [11];
- ➢ making general practice young people friendly [12];
- ➢ confidentiality of services [13];
- ➢ setting up school [14] and college [15] based sexual health services; and
- ➢ involving young people in developing local strategies [16].

Specific guidance was also developed for youth support workers [17] and social care practitioners [18] on supporting young people to access SRH advice, as well as encouraging greater involvement of young people in local policy development. Funding was also made available for teachers and school nurses to participate in a national continuing professional development (CPD) programme to improve the quality of SRE [19, 20].

Communications campaign activities, informed by academic research, included - *Sex. RUthinking about it enough? –*aimed at 13 to 17 year olds, with headline messages on resisting peer pressure to have early sex, encouraging early access to confidential services and using condoms and contraception to prevent pregnancy and STIs; articles and advice in teenage magazines provided greater detail. A free telephone helpline for young people (Sexwise) was available from 7 am to midnight, 7 days a week. A separate initiative – *Time to Talk* - encouraged parents to talk to their children about sex and relationships and was delivered through Parentline Plus, an NGO trusted by parents [6].

The strategy’s action plan also included a commissioned research programme to explore issues identified by the SEU report as requiring more understanding [21].

Progress of the strategy was monitored through the quarterly and annual conception data and through the annual reports and performance management process led by the regional coordinators and the Unit. The reach of the campaign was monitored through an annual tracking survey. Independent academic researchers were commissioned to evaluate the first 4 years’ implementation of the strategy [22].

### Mid-course review

By 2005, the national under-18 conception rate had declined by 11% but there was wide variation in progress, with local authority rates varying between a reduction of 42% and an increase of 43%. This prompted a ‘deep dive’ review of six areas, led by the Unit and the Prime Minister’s Delivery Unit^1^ [23]. Three matched areas where rates had declined and three where rates had increased were compared. Each area was visited over 2 to 3 days by a team from the Unit and experts from the IAG, and detailed interviews and focus groups were conducted with senior leaders, service managers, frontline practitioners and young people. The review found that areas with larger reductions were implementing most, if not all, aspects of the strategy, involving all relevant agencies to create a ‘whole systems’ approach, with strong senior leadership; this contrasted with areas showing increases in rates.

**Fig. 1:**
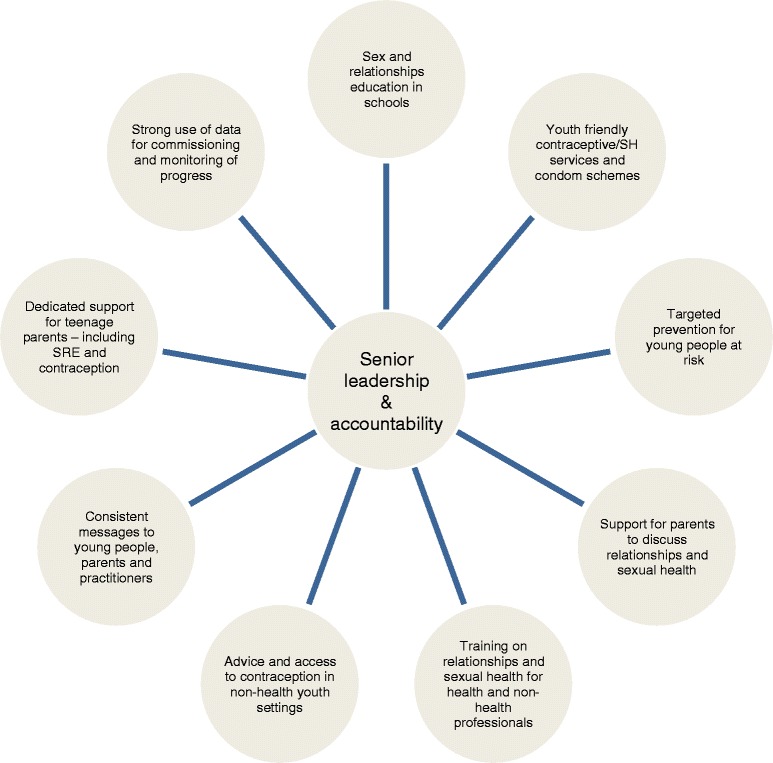
Ten key factors for effective local strategies

Drawing on the review, Government issued new more prescriptive guidance for local areas, setting out the ten key factors for an effective local strategy (Fig. 1), together with detailed local data analysis and information to strengthen targeted work with the most vulnerable young people [23]. A self-assessment toolkit was provided to help areas identify and address gaps in their local plans and strengthen local performance management [24]. Additionally, a number of areas were placed under close ministerial scrutiny, received additional support from the regional coordinators and a Department of Health National Support Team^2^, and were invited to national meetings to share good practice. Six monthly reports on progress were requested by ministers [25].

The Unit also reviewed the national campaign. A redesigned *RUthinking* campaign continued to provide universal messages for younger teenagers and a new campaign*, Want Respect?: Use a Condom*, was developed for older teenagers, with a focus on boys and those most at risk [26]. The Sexwise helpline continued to provide confidential advice and referrals to local services, with half of the calls received being from boys and young men. Campaign materials were free for local areas to use in relevant settings. Further partnerships were developed with the private and commercial sector to link the *Want Respect?* messages with brands popular with the target group [26]. The *Time to Talk* campaign for parents continued with posters and leaflets disseminated through 10,000 general practice/family doctor surgeries [27].

No additional strategy funding was provided to local areas but an increasingly supportive policy environment strengthened the traction of the new guidance and ministerial focus. New legislation [28] put a legal duty on local areas to cooperate with partner agencies and promote Every Child Matters (ECM) - a more joined up and holistic approach to improving the health, education and wellbeing of children and young people. Each area formed a Children’s Trust with a requirement to develop a Children and Young People’s Plan to meet the five ECM outcomes, which included reducing the under-18 conception rate [29]. To reflect the more integrated approach, the Unit was moved from the Department of Health to a new Children, Young People and Families Directorate in the Department of Education and Skills, which later became the Department for Children, Schools and Families. The strong partnership work continued with the Public Health Minister in the Department of Health highlighting the importance of collective responsibility for progress [19].

The reach of the strategy was also increased through other Government programmes, including

- ➢ a new public health programme which provided additional investment in sexual health improvements [30];
- ➢ the continued implementation of the all age Sexual Health and HIV Strategy [31];
- ➢ a strengthened Healthy Schools programme which required schools to deliver a planned SRE programme to achieve a National Healthy Schools Standard [32];
- ➢ an expectation of all schools to develop extended services, including health drop-ins [33];
- ➢ a new Targeted Youth Support programme to reach vulnerable young people [34]; and
- ➢ the *You’re Welcome* quality criteria for the commissioning of young people friendly health services [35].

In 2007, in addition to the new guidance and ministerial focus on improving local performance, further consideration was given to what national government action would help accelerate progress. Following an updated international research review, the decision was taken to focus on the two key areas with the strongest empirical evidence on reducing teenage conception rates: improving the provision of high quality comprehensive SRE [36], and increasing uptake and effective use of contraception [37].

From the start of the strategy there had been a sustained call to government from the Advisory Group, the Sex Education Forum and other stakeholders, to make PSHE and SRE a statutory part of the school curriculum. Two reports from the school inspection service – Ofsted^3^ - had also highlighted SRE as the weakest element of PSHE, calling for significant improvements in the quality of teaching and assessment of students’ learning [38, 39]. Statutory status was considered essential to address the inconsistent provision and unacceptable variation for young people in whether or not they received appropriate SRE. Following an influential survey of 20,000 young people led by the UK Youth Parliament [40], and a Sex Education Forum campaign [41], Government commissioned a review of SRE and PSHE, and included representatives from a wide range of organisations, including faith groups [42].

In 2008, ministers accepted the review recommendations to make SRE and PSHE statutory [43], but the legislation failed to get passed during the final legislative process in April 2010 that preceded the general election. However, the expectation of statutory status prompted some local areas to raise the priority of SRE and develop programmes and training to prepare schools.

The focus on improving young people’s access to, and effective use of, contraception was highlighted by the trend in the under-18 conception data; the decline in births to under 18 s was faster than the overall decline in conception rates implying that greater access to contraception was required [44]. To support improvements, the Department of Health secured £33 million additional funding from the government’s Comprehensive Spending Review settlement for 2008-11 [45], with the aim being to increase access to all types of contraception, but particularly to ensure that Long Acting Reversible Contraception (LARC) choices were well publicised and easily available in local areas to women of all ages, including those under 18. Funds were distributed regionally with a focus on improving access for young people, and on activities such as health professional training on LARC fitting, which would be sustainable beyond the 3-year lifetime of the earmarked funding [45]. To continue the important promotion of condoms to protect against STIs and involvement of boys and young men, guidance was published on local condom distribution schemes [46], with special attention being paid to outreach work [47].

As part of the drive to improve awareness and uptake of effective contraception, and to increase awareness and screening of STIs, a new national campaign was launched – *Sex. Worth Talking About.* The campaign was informed by a marketing review [48] which showed that the previous targeted campaigns of *Ruthinking* and *Want Respect?* had increased awareness and improved behavioural intentions towards safer sex, but that the greatest impact of a national communications campaign would be achieved by promoting and modelling a more open culture around reproductive and sexual health advice. The campaign ads showed everyday conversations about contraception and chlamydia between young people, with parents, and with professionals, on radio, cinema and TV, and were broadcast at times to reach the widest possible audience. Materials reinforcing the campaign messages were available free to local areas [49]. Further partnerships were developed with the private and commercial sector including distribution of a leaflet for parents, *Talking to your teenager about sex and relationships* [50] through 3000 independent pharmacies. Pharmacies were chosen as a non-threatening and trusted source of health advice with a presence in all local communities, including the most deprived. The campaign was launched in November 2009 but only ran for a few months before the change of government in May 2010.

The impact of these strategy activities was again strengthened by an increasingly supportive policy environment, which helped to integrate teenage pregnancy work into wider programmes for improving the health and wellbeing of children, young people and families. The under-18 conception rate was included as one of five indicators in a new national government Public Service Agreement, a new *Child Health Strategy* [51] highlighted the importance of reducing teenage pregnancy, and a national and regional team supported local implementation of the *You’re Welcome* standards. New legislation [52] which placed a duty on school governing bodies to promote pupils’ wellbeing provided an incentive for schools to review and develop their SRE and PSHE policies. The Sex Education Forum was funded to develop a resource for schools to audit pupils’ views on the extent to which SRE was seen to meet their needs [53].

Progress in reducing the under-18 conception rate continued to be included in the performance frameworks of health, social services and local government. A revised self-assessment toolkit was published with an edited data set for monitoring progress [54]. Completion of the self-assessment was included as a marker of local government commitment to teenage pregnancy in the Comprehensive Performance Assessment rating. Local government’s increasing prioritisation of teenage pregnancy was highlighted in their autonomous choice of indicators for monitoring local progress (Local Area Agreements), with the under-18 conception rate chosen by 106 of the 150 areas [45].

Visible ministerial leadership at national and regional events and media interviews provided a backdrop illustrating government’s sustained commitment to teenage pregnancy [45]. Areas with slow progress continued to receive additional support from the regional coordinators and the National Support Teams.

Further guidance was published in February 2010, informed by an updated evidence review and lessons from effective local practice [55]. The guidance included some national commitments to support further progress, including legislation for statutory SRE. However, the majority of these were not implemented due to the change of government after the general election in May 2010.

**Fig. 2:**
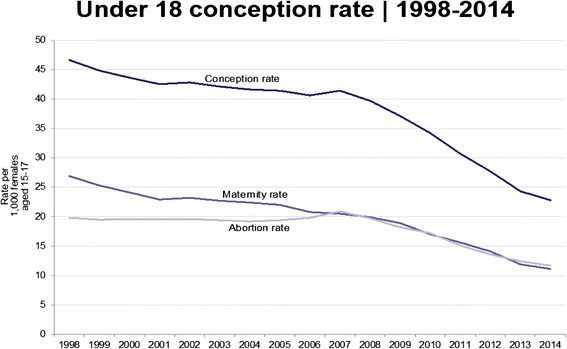
Under-18 conception, maternity and abortion rates, 1998 to 2014

The measure of progress for the strategy was the under-18 conception rate, published at national and local area levels. The latest data for 2014, published in March 2016, showed a 51% reduction in the under-18 conception rate from 1998 [56]; all local areas, including those with high deprivation, experienced reductions; and maternity and abortion rates were declining in parallel (Fig. 2). Although national data on other indices are limited, there was a doubling in the number of youth specific community contraceptive clinic sessions between 1997/8 and 2009/10 [57, 58], a large increase in the use of LARCs by under 18 s accessing contraceptive clinics [58], a significant expansion in the number of school and college based clinics providing reproductive and sexual health advice [59, 60] and an upward trend in the proportion of young people reporting school as their main source of SRE [61], which is associated with lower under-18 conception rates and STI diagnoses.

As Fig. 2 illustrates, the reduction in the conception rate accelerated after 2008. Interestingly, the downward trend continued despite the impact of the recession. During the UK recession in the early 1980s, there had been an increase in the under-20s conception – mainly maternity – rate [62]. Based on the trend in the 1980s, and the strong association between teenage pregnancy and deprivation, government scenario planning for the 2008 recession predicted a reversal of the downward trend; however, the data did not follow the anticipated pattern. Between 2008 and 2014, the conception rate fell by a further 42.5%, with both abortion and maternity rates declining. The contributory factors to this later decline are discussed in the next section.

## Discussion

As teenage pregnancy rates are influenced by a web of inter-connected factors, the strategy was necessarily multi-faceted in its approach. As such, it is not possible to identify direct causative pathways or estimate the relative contributions of each constituent part. However, an observational study reporting the long-term independent evaluation of the strategy has confirmed its impact, with the greatest effect in areas of high deprivation which received larger levels of investment [3, 63]. By studying the detailed documentation of how the strategy was designed and implemented, six key features have been identified which are considered as being fundamental to its success. Some reflections of what might have strengthened or accelerated the impact of the strategy are also briefly summarised. This analysis complements previous analysis of the strategy against the World Health Organisation (WHO) ExpandNet Framework [2].

### Six key features for success

#### Creating, and maximising, the opportunity for concerted and collaborative action

The incoming Labour Government recognised that teenage pregnancy was both a cause and consequence of inter-generational inequalities. A commitment to reduce rates, and improve outcomes for young parents, was therefore central to the government’s wider ambition to reduce social exclusion. Professional organisations and NGOs, who had long advocated for more focused action, strongly endorsed the strategy as an opportunity to improve support and choices for young people. The willingness for collaboration was harnessed by the strategy’s governance structures and legislative changes to strengthen multi-agency working and accountability. The unique spirit of positive collaboration was noted in the first phase evaluation, which described the strategy being ‘implemented with energy and enthusiasm, in an atmosphere of cooperation and consensus of those involved’ [22].

#### Developing an evidence based strategy with agreed local and national targets

The strategy was based on a careful analysis of the likely reasons behind England’s high rates informed by an international evidence review. The evidence for alternative approaches, such as abstinence-only education or withdrawal of benefits for young parents, were clearly refuted. Delegating the development of the strategy to the new Social Exclusion Unit, rather than a government department, gave the strategy credibility with stakeholders and helped ensure it was comprehensive in both its analysis and programme of action. Having a national goal to halve the under-18 conception rate and agreeing local reduction targets with each area gave the strategy a clear –albeit challenging - focus, which was easily communicated to all partners.

#### Establishing the structures and working mechanisms for effective strategy implementation

The strategy action plan provided a clear set of instructions for implementation, with guidance for all key agencies. National, regional and local structures provided the framework for implementation, which was maintained throughout the 10-year programme. The dedicated national Unit was essential for leading the strategy, with the team bringing together civil servants and respected external specialists with a wide range of varying perspectives on the topic. The inter-departmental ministerial board facilitated cross-government working and the Advisory Group was appointed to hold government to account. The national forums for NGOs and faith organisations helped sustain wider stakeholder engagement. The regional coordinators supported the local areas, organised network meetings to disseminate strategy guidance and shared good practice. Every local government area appointed a teenage pregnancy coordinator and a partnership board, and developed a strategy, which was assessed by the regional coordinator and national Unit. All areas received dedicated annual funding, conditional on establishing their board and providing an annual report on progress; this helped to maintain focus even if early results were not particular promising. Very accurate conception data were published quarterly and annually by the independent national statistics office and provided a regular and objective measurement of progress. The robustness of the data also allowed comparisons of progress between similar areas, which facilitated the important mid-course review, and helped to convince the poorer performing areas that change was indeed possible.

#### Tailoring strategy actions to reflect the findings of progress reviews

The strategy was regularly reviewed to consider any new evidence, identify strengths and weaknesses and make necessary adjustments. The comparison of high and poor performing areas in the mid-course ‘deep dive’ confirmed that if all the strategy actions were implemented, rates would come down, even in deprived areas. The subsequent more prescriptive guidance, a self-assessment toolkit for local areas, additional support, and ministerial focus provided renewed focus and commitment. Identifying that conceptions leading to maternity were declining faster than those resulting in abortions, confirmed that the vast majority of young people were not choosing early parenthood. This prompted additional investment to improve access to effective contraception, which was associated with an increase in under-18 young women choosing one of the LARC methods. The actions resulting from both reviews are likely to have contributed substantially to the accelerated decline in the conception rate after 2007.

#### Embedding the strategy in wider government programmes

From the start, the strategy actions were integrated with government policy and programmes aimed at improving health, education and economic outcomes for young people. Including the target in the cross-government national Public Service Agreements and as a performance indicator for local government and their health partners anchored the strategy in mainstream performance management. Moving the Unit from the Department of Health to the new Children, Young People and Families Directorate signalled that the strategy’s goal was integral to improving wider outcomes for children and young people. The new legislation which obliged local government and their partner agencies to work cooperatively was essential in maintaining focus and maximising the range of skills and resources available in the local areas.

#### Providing and maintaining leadership throughout the strategy

Government leadership was key in putting teenage pregnancy high on the national agenda, reflected by the launch of the strategy by the then Prime Minister. It was also critical for sustaining the priority over the 10-year period, even though early progress was slow and some media commentators were claiming the strategy had failed. By being convinced by evidence that change in complex social phenomena takes time empowered the policy-makers to not seek quick results. After the mid-course review, the visible ministerial presence and direct engagement with local areas acted as a catalyst for renewed commitment. Local leadership from elected councillors and senior officials was also important in challenging a deeply held view in some quarters that high rates were inevitable and helping to maintain motivation. National and local leaders were also supported by the Advisory Group, which provided independent expert challenge to media criticism and offered constructive advice to ministers and local areas.

### What might have been done differently to strengthen the strategy?

The major shortcoming of the strategy’s implementation was the failure to make sex and relationships education a statutory part of the curriculum from the start, with the government’s eventual decision to change the status taken too late to complete legislation before the 2010 election and change of administration. Although local areas and individual schools made substantial progress, having no statutory lever to raise the priority in all schools was a significant barrier to securing universal provision. Statutory status would not have automatically led to improved quality but it could have helped increase the supply of trained educators, encouraged sufficient curriculum time, and led to improved programmes of study with clear learning outcomes, as well as inclusion in school inspections. The impact of statutory status would have taken time to reach all young people, but it is likely that it would have contributed to more rapid progress in reducing conception rates. It would have also enshrined young people’s entitlement to SRE in mainstream government policy, and helped to sustain the foundations of prevention beyond the lifespan of the strategy.

Other actions to address factors that became apparent as the strategy unfolded may have strengthened or speeded up its impact. They include: issuing more prescriptive guidance from the start for local areas to develop their strategies, with clear ‘must do’ actions for each agency, and stronger performance management of implementation; having a stronger focus in the early national campaigns on increasing knowledge and uptake of effective contraception, rather than just condoms; developing an annual survey of young people to monitor the strategy’s impact on improving SRE, access to contraception and confidence in asking for advice, which would provide a national data set and be available for local areas to track impact on their local population; and more proactive work with the media to highlight the consensus between parents, young people and professionals for improving SRE and access to contraception. A national media strategy to highlight the consensus and lead a well-informed discussion might have helped counter some unhelpful and misinformed media reports and promote the more open culture which, evidence suggests, is fundamental to helping young people to discuss relationships and sexual health and ask for advice without stigma or embarrassment. These factors are discussed in more detail elsewhere [64].

## Conclusion

As reducing teenage pregnancy rates is a complex issue it is important to avoid a reductionist approach to explanation. The evidence based, multi-faceted and multi-level approach, implemented with sufficient time and facilitated by a government committed to change were all instrumental in achieving the success. The learning from the strategy remains relevant for UK policy and practice as, despite the substantial decline, England’s teenage birth rate remains higher than that in other Western European countries. The key features contributing to the strategy’s success, together with the detail of the intervention - often missing from research papers - also provide important lessons for governments and policy makers in other countries seeking to reduce teenage pregnancy rates.

## Footnotes

1 The Labour Government established the Prime Minister’s Delivery Unit in 2001 to monitor progress and strengthen capacity to deliver its key priorities. The unit was closed after the change of government in 2010.

2 The National Support Teams were established by the Department of Health in 2006 to work directly with health and local government to support them to deliver their public health priorities. The Teenage Pregnancy National Support Team involved members of the Advisory Group, the Unit and the regional coordinators.

3 Office for Standards in Education, Children’s Services and Skills is a non-ministerial department of the UK government, reporting to Parliament via the Department for Education.
